# Plant pathogenesis: Toward multidimensional understanding of the microbiome

**DOI:** 10.1002/imt2.129

**Published:** 2023-07-16

**Authors:** Tianxing Lv, Chengfang Zhan, Qianqian Pan, Haorong Xu, Hongda Fang, Mengcen Wang, Haruna Matsumoto

**Affiliations:** ^1^ State Key Laboratory of Rice Biology & Ministry of Agricultural and Rural Affairs Laboratory of Molecular Biology of Crop Pathogens and Insects Zhejiang University Hangzhou China; ^2^ Key Laboratory of Biology of Crop Pathogens and Insects of Zhejiang Province, Institute of Pesticide and Environmental Toxicology, College of Agriculture and Biotechnology Zhejiang University Hangzhou China; ^3^ Global Education Program for AgriScience Frontiers, Graduate School of Agriculture Hokkaido University Sapporo Japan

**Keywords:** disease epidemics, multitrophic interaction, pathobiome, plant microbiome, plant pathogenesis

## Abstract

Single pathogen‐targeted disease management measure has shown drawbacks in field efficacy under the scenario of global change. An in‐depth understanding of plant pathogenesis will provide a promising solution but faces the challenges of the emerging paradigm involving the plant microbiome. While the beneficial impact of the plant microbiome is well characterized, their potential role in facilitating pathological processes has so far remained largely overlooked. To address these unsolved controversies and emerging challenges, we hereby highlight the pathobiome, the disease‐assisting portion hidden in the plant microbiome, in the plant pathogenesis paradigm. We review the detrimental actions mediated by the pathobiome at multiple scales and further discuss how natural and human triggers result in the prevalence of the plant pathobiome, which would probably provide a clue to the mitigation of plant disease epidemics. Collectively, the article would advance the current insight into plant pathogenesis and also pave a new way to cope with the upward trends of plant disease by designing the pathobiome‐targeted measure.

## Highlights

- The resident microbiota is a hidden game changer in plant health and disease.

- The invasive pathogen can hijack certain members of the resident microbiota to form partnerships within the reassembled community, which is currently conceptualized as the pathobiome.

- The pathobiome facilitates the pathological process of host plants, from the onset of the disease to its progression, by coordinating complex interactions at multiple scales.

- The pathobiome‐targeted approach may pave a new way to address the increasing trends of plant diseases in the face of global change.

## Graphical abstract

Understanding plant pathogenesis faces the challenges of the emerging paradigm involving the plant microbiome. In this article, we highlight the detrimental actions mediated by the pathobiome at multiple scales and further discuss how natural and human triggers result in the prevalence of the plant pathobiome, which would probably provide a clue to mitigation of the plant disease epidemics.

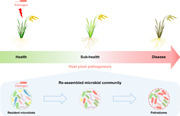

## INTRODUCTION

The plant microbiome conventionally refers to the resident microbiota as well as the whole spectrum of molecules they produce in the host plants [1, 2]. The native plant microbiota mediates an interaction network at multiple scales, ranging from intramicrobiome interactions to multitrophic interactions with their host plants, exogenous microbes, and insects [3, 4]. Such complex interactions have so far gained both scientific and public interests because they determine the status of the host plants, with an implication for agricultural production and global food supply [5, 6]. In the efforts researchers previously made, the plant microbiome has been highlighted mostly due to their contribution to the improvement of the fitness and health of the host plants [7, 8, 9, 10]. The disease‐preventing members of the resident microbiota recently coined as soterobionts are known to function as a defensive layer against pathogen invasion [11, 12, 13, 14], which is consistent with the hologenome‐derived holobiont theory [15].

**Figure 1:**
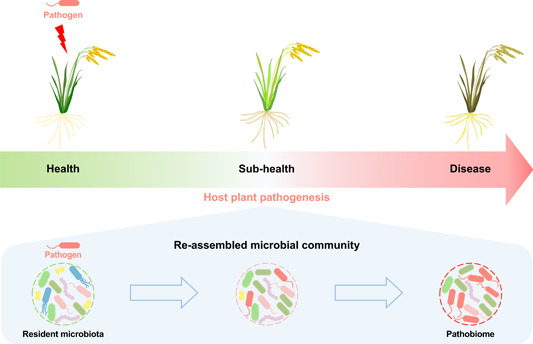
The schematic representation of the pathobiome paradigm in the plant pathological process. Alongside the pathological process of host plants, certain members of the resident microbiota can be manipulated by the invasive pathogen, forming a partnership within the reassembled community. This ultimately results in the development of a unique microbial community that corresponds to the disease status of the host plants. The microbial consortia associated with host pathogenesis, present from disease onset to progression, are collectively and contemporarily conceptualized as the pathobiome.

However, some native members in the resident microbiota can also be potential pathogens and an accumulation or change in the relative abundance of these members in their hosts can lead to disease onset or greater severity [16, 17], and some of these members even can be manipulated by the invasive pathogen to form the partnership along with the pathogenesis process (Figure 1) [18, 19]. The association of the host–resident microbiota with host pathogenesis has already aroused a conceptual controversy in hologenome [20], and the host pathogenesis‐associated microbial consortia, presenting from disease onset and progression, are contemporarily conceptualized as pathobiome (Figure 1) [21, 22]. The emerging evidence has already shown that the inoculation of the microbiota from the leaves of diseased plants into healthy plants could result in leaf damage [23], suggesting the pathobiome acts opposite to soterobionts, but the causality between the pathobiome and host pathogenesis remains largely elusive in plants. At present, the pathobiome and the associated mechanistic insight are scarcely explored in plants, in particular, the unfolding actions of the pathobiome in the onset and development of diseases require in‐depth investigation.

Here, we present the controversy in the plant microbiome as well as the emerging challenges in the studies of plant microbiome‐associated pathogenesis. In the pathobiome‐included plant pathogenesis paradigm (Figure 1), we discuss the detrimental actions mediated by the plant pathobiome at multiple scales, which underlies from the onset to the progression of plant disease. We aim to highlight that the potential negative impacts of the resident microbiota on plant health should not be overlooked, and further holistic insight into the microbiota‐associated interaction needs to be expanded by the characterization of the pathobiome as well. We further discuss how natural and human triggers of the plant pathobiome prevalence act under the scenario of global change, which is possibly a key to the prevention of the plant pathobiome assembly. Collectively, this article would advance the current understanding of plant pathogenesis and offer new opportunities to cope with the upward trends of plant disease epidemics by developing pathobiome‐targeted measures.

## MICROBIAL INTRA‐ AND INTER‐SPECIES SIGNALING SYSTEMATICALLY AFFECTS PLANT PATHOGENESIS

The rapid advancement of multiomics has significantly deepened our understanding of the structural and functional diversity of the plant microbiota, and a growing awareness of pathogenesis has evolved from the “one pathogen‐one disease” paradigm to the pathobiome concept [19]. Many diseases previously attributed to one pathogenic agent are likely to be the result of interactions among/between multiple microbial taxa and their host plants. While the understanding of the roles of the plant soterobionts reveals the defense against pathogens, the mechanistic insight into the plant pathobiome‐associated interactions is important for revealing the process that governs the outcome of pathogen infection in the host plants.

Establishment of the mutualistic relationships among the invasive pathogen and the native potentially pathogenic microbial members has been hypothesized to negatively affect the homeostasis of vegetative and reproductive organs, drive the infection outcome, and result in disease progression [19, 24]. Such microbial mutualistic relationship is generally concealed within the complex intramicrobiome interaction throughout the below‐to‐above compartments from intraspecies to interspecies and interkingdom [25, 26, 27], despite how the plant pathobiome members cooperatively interact to establish such relationships are still not well understood.

Chemical communication serves as a ubiquitous way to drive the interactions within one microbial species. As a language that individual bacterium use to communicate with each other belonging to the same species, a diverse array of small molecules shape the intraspecies behavior and functions for their survival and adaption in the hosts [28]. Quorum‐sensing (QS) was the first identified bacterial intraspecies signaling system, which enables bacterial cells to chemically sense the density of the surrounding population and regulate various physiological activities such as motility, biofilms, secondary metabolism, and virulence in a cell density‐dependent manner [29, 30, 31]. Interestingly, such intraspecies communication is also observed in fungal communities [32]. Similar to bacteria, fungi also mimic quorum regulation to coordinate behaviors from the individual level to the population level involved in pathogenicities, such as germination, colony morphogenesis, sporulation, and biofilm formation [33, 34].

**Figure 2:**
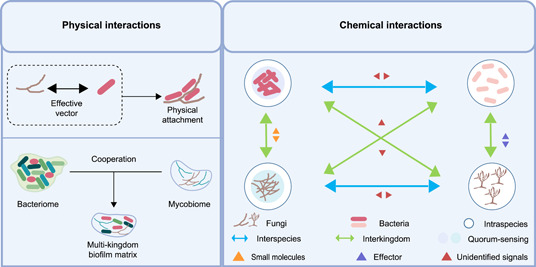
Intramicrobiome interaction models involved in plant pathogenesis. Establishment of the mutualistic relationships between the invasive pathogen and the native potentially pathogenic microbial members in the host plants has been hypothesized to impair the homeostasis of plant vegetative and reproductive organs, drive the outcome of pathogen infection, and result in disease onset and progression. Such microbial mutualistic relationship is hidden in the complex intramicrobiome interaction throughout the below‐to‐above compartments from intraspecies to interspecies and interkingdom and driven by physical and chemical interactions mediated with an array of molecules, such as small molecules, effectors, and unidentified signals.

Indeed, intraspecies communication in situ is largely impacted under a more sophisticated network (Figure 2), in which the diverse bacterial taxa resident in the same eco‐niche of the host plants mediate interspecies interactions [35]. Quorum quenching (QQ) has been previously characterized as ubiquitous interspecies interaction employed by symbiotic bacteria to interfere with the QS of phytopathogenic bacteria [36, 37]. For instance, a wide array of commensals such as *Microbacterium testaceum* from potato leaves have been found to interfere with the *N*‐acyl‐homoserine lactone (AHL)‐based QS of phytopathogens *Pectobacterium carotovorum* via QQ [38]. The further cloning of the *aiiM* gene from *M. testaceum* indicates that AiiM works as an AHL lactonase to catalyze AHL ring opening, and the expression of AiiM in the *P. carotovorum* reduces pectinase activity and attenuates soft rot symptoms on potato [39]. As a controversy, the establishment of the mutualistic relationship has been reported to be a prevalent principle within the natural microbial communities, in which bacteria typically form close mutualistic loops resulting in indirect benefits to all species involved [40, 41]. For instance, gut microbiota‐derived sources of carbon and nitrogen could be exploited by invasive bacterial pathogens as nutrients and regulatory signals to promote their own growth and virulence in mammals [42]. It has recently shown that the rhizosphere‐inhabiting beneficial bacterium *Pseudomonas fluorescens* can be converted into a pathogen while it was transferred with the disaggregated effector arsenal from the phytopathogenic bacterium *Pseudomonas syringae* PtoDC3000, which highlights the ecological scale implication of the intraspecies communication on the plant pathogenesis [43]. However, it still remains largely unanswered how the invasive pathogen establishes mutualistic communication with certain resident microbial members to promote disease progression. For understanding the group behavior involved in pathobiome‐coordinated infection in host plants, further efforts are expected to explore unidentified signaling molecules and the hidden regulatory systems governing pathogenicity beyond QS.

## MICROBIAL INTERKINGDOM INTERACTION IN PLANT DISEASE PROGRESSION

In plants, intramicrobiome interaction is not limited to the intra‐ and inter‐species level but is also featured by interkingdom interactions [44]. Within these heterogeneous microbial communities, bacteria, and fungi influence each other directly and indirectly via a way known as bacterial–fungal interactions (BFIs) [45, 46], majorly including physical interactions and chemical interactions (Figure 2).

Investigation of the specific cooperation mechanism of the assembled pathobiome in rice has further shown that *Burkholderia glumae* (Bg), one of the causal agents of bacterial panicle blight, could physically attach to *Fusarium graminearum* (Fg) to promote its survival and dispersal and consequently, the disease progression. Bg can facilitate Fg occupation in rice heads by increasing deoxynivalenol production and disease severity [47]. While this study has demonstrated that bacterium utilizes fungus as an effective vector to facilitate its infection and expansion in the host plant, less is known about how fungi physically benefit from bacterium during the mixed infection. Nevertheless, the clinical evidence suggests that the pathogenic mycobiome can benefit from bacteriome through the formation of the multikingdom biofilm matrix, which functions as a physical barrier to protect against the host and antimicrobial insults and advance the infection [45, 48].

Serving as a major track to mediate swift chemical interactions, the production of small molecules, such as volatile organic compounds (VOCs), is highly conserved in microbes and influences the physiology and even virulence traits of the interacting members [49]. It has been shown that the volatiles emitted by *Fusarium culmorum* would significantly change the motility of two bacteria *Collimonas pratensis* Ter291 and *Serratia plymuthica* PRI‐2C, which suggests that the volatiles might act as signaling molecules for attracting bacteria and further are likely to achieve co‐infection of fungi and bacteria [50]. In addition to VOC‐mediated interactions, more complicated patterns have been demonstrated between the BFI members during the disease progression in various host plants. A pioneering study conducted in the plant rhizosphere demonstrated that the soil‐borne fungal pathogen *Verticillium dahliae* releases a virulence effector VdAve1 to improve its colonization in tomato and cotton by inhibiting most plant‐associated beneficial bacteria, but some potentially pathogenic members such as *Pseudomonas corrugate*, *Ralstonia* sp., and *Serratia* sp. exhibit adaptability, suggesting the formation of the pathobiome along with the successful infection of *V. dahlia*, despite the unresolved mechanism underlying this typical interkingdom communication [51]. Similarly, bacteria have evolved an array of nanomachine channels known as bacterial secretion systems to deliver effectors for interkingdom interaction [52, 53, 54], but their role in manipulating fungal pathogenicity on host plants remains unexplored. The characterization of intracellularly released chemical molecules, ranging from small molecules to macromolecules such as effectors, would be instrumental in revealing the landscape of BFI‐mediated plant disease progression.

These findings have illuminated the importance of interkingdom cooperation in plant pathobiome actions (Figure 2), but the current understanding is still limited. It is largely unknown whether and how the microbial‐derived signaling molecules, such as chemical small molecules as well as larger effectors, drive and maintain the cooperation within the pathobiome for disease onset and progression. In addition to the recently prominent investigation of the pathobiome in various plant models at the composition level [55, 56, 57], a comprehensive understanding of the complex cooperation between the primary disease‐causal agents and other potentially pathogenic members is essential for advancing the development of the pathobiome component‐targeted antidisease strategies.

## MULTISCALE INTERACTIONS UNDERLYING THE PATHOBIOME‐RESPONSIBLE PATHOGENESIS

**Figure 3:**
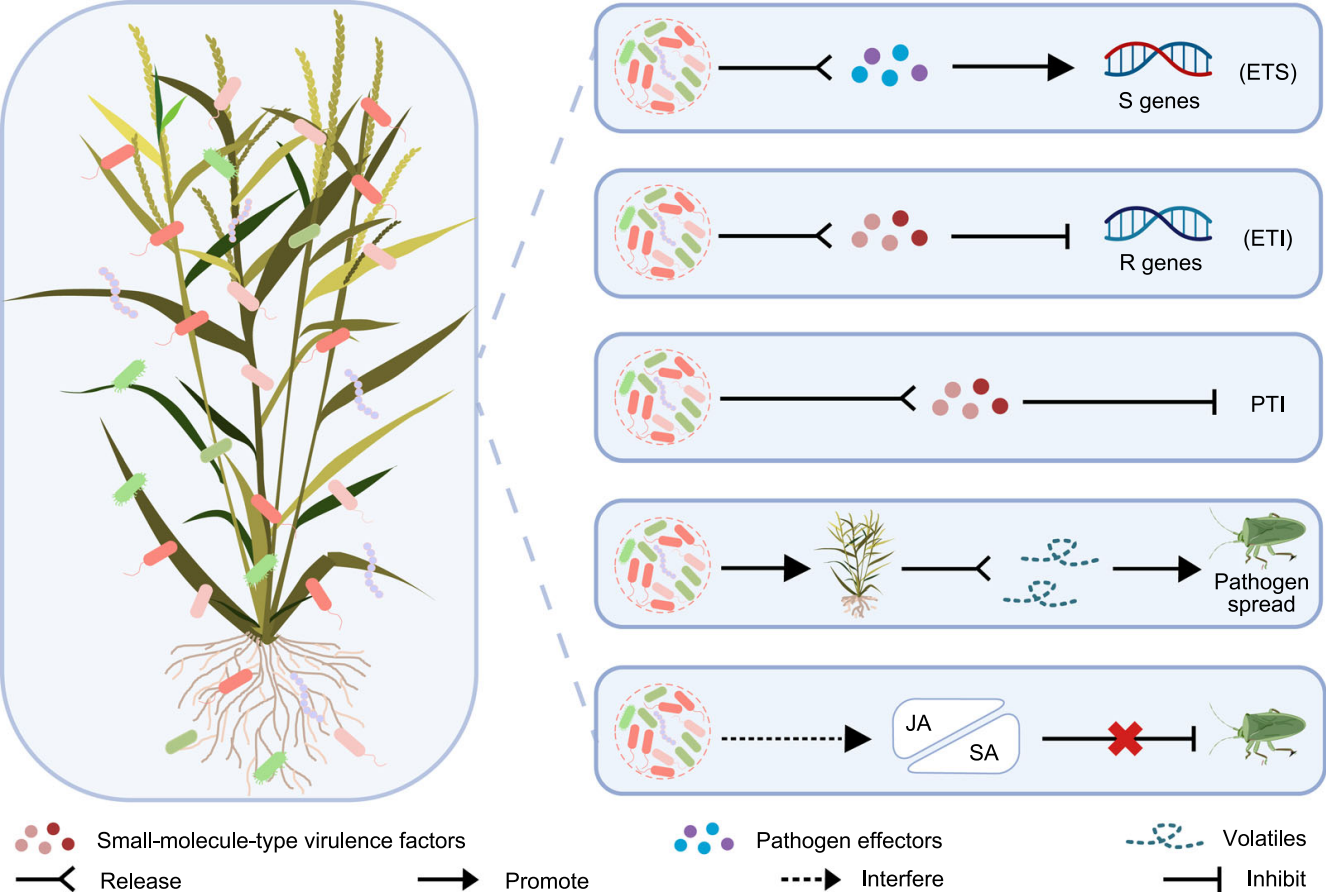
The pathobiome‐coordinated multiscale actions. The pathobiome‐mediated interaction is omnipresent at multiple scales including the plant–microbe system, microbe–insect system, and the multitrophic network involving both plants and insects, which governs the deleterious actions on the host plants. In the binary interaction model of plants and microbes, the pathobiome members are capable of releasing the active macro‐molecules such as effectors to induce host susceptibility by manipulating the traits controlled by the S genes. Various small‐molecule‐type virulence factors are also deployed to disable the plant's innate immunity‐based defense and further drive the infection to move toward disease progression. Moreover, the plant pathobiome members also participate in promoting disease epidemics by manipulating the multitrophic interactions. For instance, the pathobiome member stimulates the emission of insect‐attractive host volatiles to manipulate the host attraction to herbivore vectors for the spread of the whole pathogenic microbial community or influences the cross‐talk between the JA and SA to compromise defense against herbivores. ETI, effector‐triggered immunity; ETS, effector‐triggered susceptibility; JA, jasmonic acid; PTI, PAMP‐triggered immunity; R genes, resistance genes; S genes, susceptible genes; SA, salicylic acid.

In addition to intramicrobiome interaction, the pathobiome‐mediated interaction is omnipresent at multiple scales [58, 59], including the plant–microbe system, microbe–insect system, and the multitrophic network involving both plants and insects (Figure 3). While host plants establish close relationships with the soterobionts to defend against pathogens, these potential pathobiome members in turn collaborate to counteract the plant genetics‐governed alliance. In the quadruple mutant *mfec* (*min7/fls2/efr/cerk1*) of *Arabidopsis*, the leaf endophytic microbiota of a Firmicutes‐rich community shifted into a Proteobacteria‐rich community along with leaf necrosis and/or chlorosis phenotypes under high humidity condition. Interestingly, the bacterial community transplantation experiments demonstrated that wild‐type Col‐0 plants remained healthy when inoculated with leaf endosphere‐derived SynCom^Col‐0^, whereas dysbiotic symptoms appeared in the presence of *mfec* leaf‐derived SynCom^mfec^. The underlying mechanism by which *mfec* plants produced a dysbiotic bacterial community was distinctly associated with the deficiencies in pattern‐triggered immunity and the MIN7 vesicle‐trafficking pathway [23]. These findings provide compelling evidence for the causality between the pathobiome and the disease onset, but how the pathobiome members integrate their actions toward the host plants is still unknown.

Plant receptors, known as pattern recognition receptors (PRRs), play a crucial role in recognizing microbial molecules (microbe‐ or pathogen‐associated molecular patterns, MAMPs, or PAMPs), such as lipopolysaccharides, flagellin, elongation factor‐Tu, lipoproteins, peptidoglycans, chitin, lipopolysaccharide, triacyl lipopeptides, lipoteichoic acid, and diacyl lipopeptides, to trigger the innate immune response [60, 61]. PAMP‐triggered immunity (PTI) and effector‐triggered immunity (ETI), as the two layers of plant innate immunity, potentiate each other to strengthen plant defenses [62]. The mutualistic relationship of the pathobiome members is not only featured by the intramicrobiome cooperation but also benefits from the integration of their distinctive pathogen effectors‐assisted impacts, which can rapidly evolve to overcome ETI by evading recognition of R proteins, leading to effector‐triggered susceptibility (ETS) [63]. Effectors produced by different members of the plant pathobiome may target various plant factors encoded by susceptibility genes (S genes) to magnify the ETS of the host plants (Figure 3), such as the manipulation of entry, the acquisition of nutrients, the suppression of defenses, and the translocation of bacterial proteins [2]. Moreover, conventional defense strategies based on plant innate immunity remain ineffective when confronted with highly virulent small molecule‐based virulence systems employed by bacterial phytopathogens [5]. The pathobiome members may arm with diverse small‐molecule‐type virulence factors to subvert plant's innate immunity‐based defense strategies (Figure 3). These findings could explain the limitations of the conventionally one pathogen‐targeted approach, and more profoundly suggest that the integrated management of pathobiome members associated with the disease would lead to a breakthrough in the insufficient efficacy in the field.

The binary interaction model of insects and the associated microbiota reveals that the microbes inhabiting in herbivores can favor or improve the behavior and fitness of the hosts by manipulating hormone levels and subverting the plant defense, despite the insufficient understanding at the multitrophic level [64, 65]. It is known that a wide range of insect herbivores show a preference for attacking plants' buds, leaves, and especially flowers, and the peak incidence occurs at the flowering period in some cases, leading to a significant reduction in fruiting and final yield. Insect herbivores' survival and fitness are essentially dependent on the host plant selection in nature, in which host‐derived volatiles are the major components for the attraction of insect herbivores [66]. Interestingly, a tritrophic interaction study demonstrated that the plant pathogen *Candidatus* Liberibacter asiaticus could enhance the release of volatiles in citrus, which contributed to the host attraction of the bacterium's insect vector *Diaphorina citri* [67]. This study revealed the exquisite modulation of herbivore–plant interactions by the plant pathogen, suggesting that the specific members of the plant pathobiome possibly employ a similar strategy to promote their spread and disease epidemics via manipulating the host attraction to insect herbivore vectors (Figure 3), despite the unidentified regulatory pathway by which the pathobiome stimulates emission of the insect‐attractive host volatiles.

Besides, emerging evidence has shown that the native microbiota could compromise defense against insect herbivores by influencing the cross‐talk between the phytohormones jasmonic acid (JA) and salicylic acid (SA) [68]. The signaling between JA and SA is known to be antagonistic and the signaling trade‐offs in plant defense have important ecological consequences in nature that may be a general mechanism by which the pathobiome member indirectly influences the ecology and evolution of insect herbivores and vice versa. For instance, certain pathobiome member may suppress JA signaling and the related defense against insect herbivores by inducing SA signaling (Figure 3). Hence, the characterization of the plant pathobiome members that direct the deleterious actions at multiscale will provide an alternative way of pesticides to disorder the herbivore behavior. The future insight into the multitrophic interactions in the plant–insect–microbe system serves as an important basis for the novel strategies to break both pathobiome‐responsible pathogenesis and insect pest damage.

## TRIGGERS BEHIND THE PLANT PATHOBIOME PREVALENCE

A critical question remains unanswered as to what and how a certain circumstance triggers the plant pathobiome prevalence. Global change has been predicted to continue with more drastic trends since the last century, and its impact on the plant microbiome has recently received attention due to the cascading effects on plant productivity, biodiversity, and ecosystem functioning [69, 70]. Given that the plant disease epidemics arise under the scenario of global change [71], it is time to pay attention to the implication of global change on the prevalence of the plant pathobiome.

**Figure 4:**
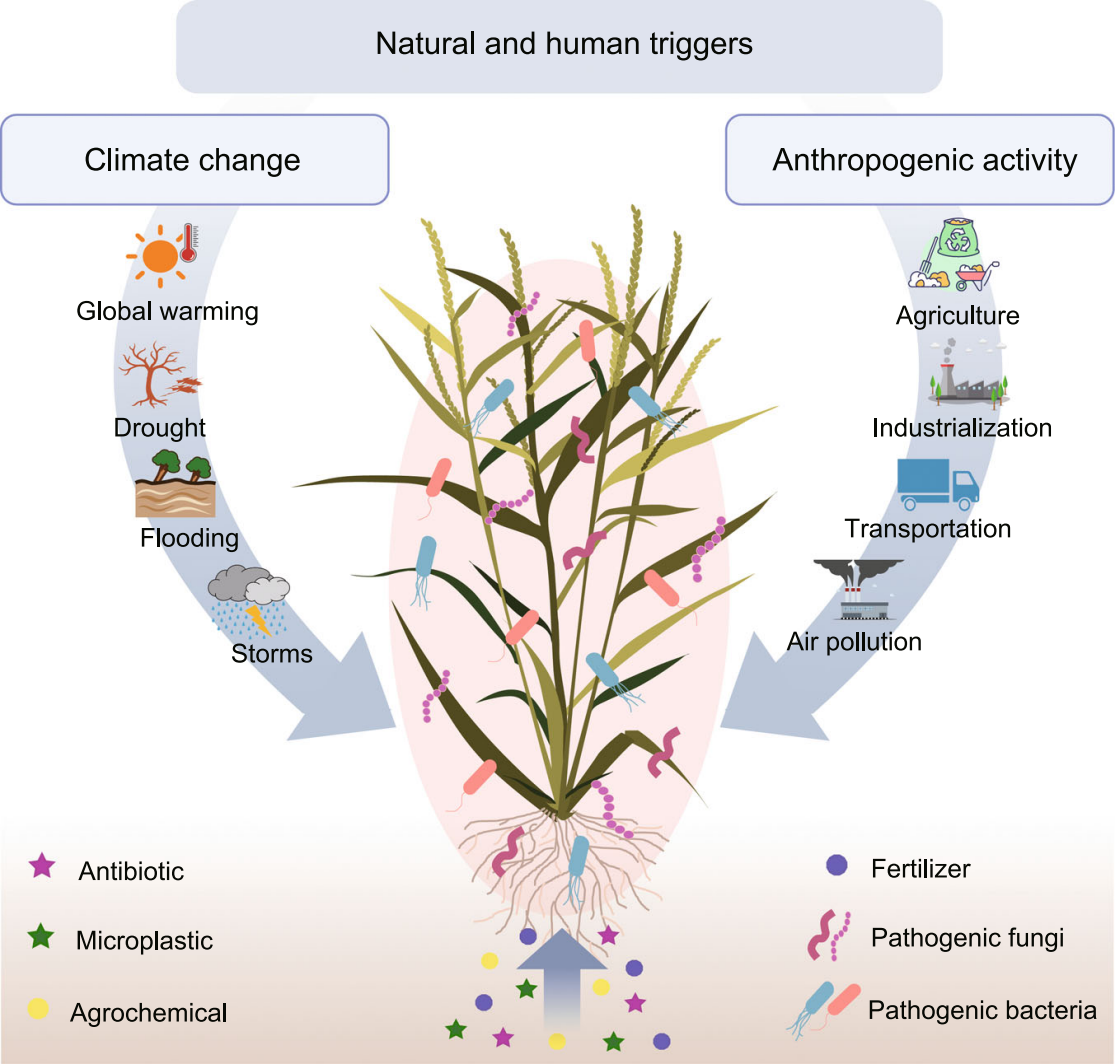
Potential triggers responsible for the plant pathobiome prevalence. Given that plant disease epidemics arise under the scenario of global change, a series of natural and human triggers responsible for the prevalence of the plant pathobiome are highlighted. An increasing trend of plant disease outbreaks has been observed with climate change, such as global warming, and increased frequency of extreme weather events (e.g., drought stress, flooding, and storms). Global climate change has been hypothesized to trigger the plant pathobiome prevalence by promoting the assembly of the pathobiome member. As drivers of global change in the current human‐dominated epoch, anthropogenic activity (e.g., agriculture, industrialization, energy production, and transportation) and anthropogenic activity‐associated environmental pollutants further strengthen plant microbiome prevalence through a “vacuum effect,” which enable the nonnative potentially pathogenic microbes from the surrounding environment settling in below‐to‐above compartments of host plants.

Climate change is a natural hallmark of global change and the Earth is experiencing dramatic climate changes such as global warming and increased frequency of extreme weather events (Figure 4). An increasing trend of plant disease outbreaks has been observed with global warming. Mechanistic investigation has shown that warm temperature negatively affects the innate immunity of plants by compromising JA‐regulated basal resistance, thereby promoting *Magnaporthe oryzae* infection in rice [72]. Going beyond the binary interaction model of the host plants and one pathogen, the long‐term effects of moderate surface warming (+2°C) on the composition and diversity of the leaf microbiota of *Galium album* were investigated. The results show a reduction of the relative abundances of beneficial bacterial taxa such as *Sphingomonas* and *Rhizobium*, along with the enrichment of a group of potentially pathogenic taxa (e.g., *Enterobacter*, *Erwinia*, and *Acinetobacter*) [73]. Additionally, a significant loss of diversity and the disruption of the structure of the resident microbiota under drought stress have been demonstrated, whereas the taxonomic information and potential impact from the drought stress‐enriched microbial member are insufficiently discussed [74]. More efforts need to be further made to clarify the mechanisms underlying the climate change‐triggered plant pathobiome prevalence.

It is also remarkable that anthropogenic activity (e.g., agriculture, industrialization, energy production, and transportation) is a driver of global change in the current human‐dominated epoch, which has a huge impact on terrestrial ecosystems [75, 76, 77, 78]. It is still not well understood how anthropogenic activity directly triggers the plant pathobiome prevalence, but the serious occurrence of anthropogenic activity‐associated environmental pollutants in various plant‐grown habitats has emerged as a key clue in a series of case studies recently completed (Figure 4). At the air interface of the phyllosphere, air‐borne particulate matter, ozone, carbon dioxide, sulfur dioxide, and nitrogen oxides destroy the composition and decrease the phyllosphere microbial community diversity (Figure 4), as well as further enhance the plant–pathogen infection and trigger cascading effects on plant diseases [79, 80, 81, 82]. Because the phyllosphere is typically characterized as a nutrient‐scarce environment, the diversity of the phyllosphere microbiota is more sensitive to changes in mineral availability [83]. It has been shown that high levels of available phosphorus can deplete plant‐beneficial microbes but increase pathobiome abundance [56]. In the water and soil, the prevalence of antibiotics and microplastics has been found to increase the abundance of potential pathogens (Figure 4), which are spatially co‐located with an increased abundance of antibiotic resistance genes in the rhizosphere [84]. Remarkably, the increased inputs of agrochemicals and fertilizers cause uncertain impacts on the structure and function of the resident microbiota in both rhizosphere and phyllosphere and reversely allow the nonnative potentially pathogenic microbes from the surrounding environment to colonize the below‐to‐above niches of host plants through a “vacuum effect” [85]. On the contrary, sustainable agricultural management, such as organic farming, not only promotes the enrichment of plant growth‐promoting bacteria in plants but also reduce the risk of pathobiome prevalence by decreasing the abundance of potential pathogens [86]. In summary, answering how these natural and human triggers influence the prevalence of plant pathobiome is a fundamental step to prevent the assembly of the plant pathobiome before they initiate pathogenic actions.

## CONCLUSION AND FUTURE PERSPECTIVES

Current disease prevention is anchored in existing frameworks, such as Koch's Postulates, and is thus predominantly dependent on chemical fungicide application that is designed to prevent single pathogens in agricultural production [87]. However, single pathogen‐targeted prevention has gradually fallen short of control efficacy in the field. Emerging evidence shows the involvement of the pathobiome in plant pathogenesis, suggesting that the onset and progression of plant disease are not simply dominated by a single pathogen but rather the pathobiome‐coordinated interactions at multiscale. Although we still stand at the dawn of the pathobiome‐included plant pathogenesis paradigm, a promising solution to the current bottleneck in disease prevention is guaranteed, especially when further efforts are made to not only reveal the specific member in the pathobiome at the taxonomic level but also molecular understanding of the mechanisms underpinning the assembly and multiple actions of the pathobiome to direct the infection toward disease.

## AUTHOR CONTRIBUTIONS

Haruna Matsumoto, Mengcen Wang, and Tianxing Lv provided the original idea. Tianxing Lv, Haruna Matsumoto, and Mengcen Wang wrote the manuscript. Chengfang Zhan, Qianqian Pan, Hongda Fang, and Haorong Xu contributed to the collection of the case studies. Mengcen Wang and Haruna Matsumoto critically revised the manuscript.

## CONFLICT OF INTEREST STATEMENT

The authors declare no conflict of interest.

## Data Availability

This manuscript does not generate any code or data. Supporting Information (figures, tables, scripts, graphical abstract, slides, videos, Chinese translated version, and update materials) may be found in the online DOI or iMeta Science http://www.imeta.science/.
